# The effect of topical thiocolchicoside in preventing and reducing the increase of muscle tone, stiffness, and soreness: A real-life study on top-level road cyclists during stage competition

**DOI:** 10.1097/MD.0000000000007659

**Published:** 2017-07-28

**Authors:** Marco Gervasi, Davide Sisti, Piero Benelli, Eneko Fernández-Peña, Cinzia Calcabrini, Marco B.L. Rocchi, Luigi Lanata, Michela Bagnasco, Andrea Tonti, Stocchi Vilberto, Piero Sestili

**Affiliations:** a Department of Biomolecular Sciences, University of Urbino Carlo Bo, Urbino, Italy; b Department of Physical Education and Sport, University of the Basque Country UPV/EHU, Vitoria-Gasteiz, Spain; c Medical Department, Dompé Farmaceutici S.p.a., Milano; d Technical Committee of LCP Cycling Professional League, Rome, Italy.

**Keywords:** contracture, elasticity, stiffness, thiocolchicoside, tone, top class road cyclists

## Abstract

In professional road cyclists, the majority of overuse injuries affect the lower limbs and are mostly represented by contractures or muscle shortening, characterized by an increase of tone and stiffness and a variation of elasticity. Treatment and prevention of these specific conditions may include physical, supplementary, and pharmacologic support. The aim of this real-life study was to determine: first, the alterations of tone, stiffness, elasticity, and soreness of rectus femoris (RF) and biceps femoris (BF) in top class cyclists engaged in 3 multistage races, and second, whether any variable in the management of the athletes may affect the prevention and/or reduction of such alterations.

Twenty-three professional cyclists competing in 3 international, cycling stage races were assessed. Athletes could receive, upon the approval of the medical staff, physical, dietary, and/or pharmacological management which could include treatments with topical over-the-counter myorelaxants to prevent and/or reduce muscle contractures. MyotonPro was used to daily measure tone, stiffness, and elasticity in RF and BF in relaxed and contracted state after every stage. In parallel, BF and RF soreness was also assessed with a Likert scale.

All athletes received the same general massage management; none of them received dietary supplements; some of the athletes were treated with a topical myorelaxant thiocolchicoside (TCC 0.25%) foam 3 times daily. TCC was identified as the only variable able to affect these muscle parameters in the cyclists. Tone, stiffness (regardless of the state), and soreness significantly increased over time either in BF or RF in all athletes. In the group of athletes that used TCC (n = 11; TCC+) the increase in tone, stiffness, and soreness was significantly lower than in the group not receiving TCC (n = 12; No-TCC). Elasticity varied coherently with tone and stiffness.

A very intense and protracted sport activity increases muscular tone, stiffness, and soreness over time. Topical TCC foam significantly attenuates these alterations and might represent an efficient strategy both to prevent and manage contractures and their consequences in professional cyclists as well in athletes from other disciplines involving similar workloads.

## 1 Introduction

During training and competitions, cyclists are subjected to both traumatic and overuse injuries: in particular, the incidence of overuse injuries in recreational cyclists over a 1-year period may be higher than 85%.^[1,2]^ A history of sport-related overuse injuries to inferior limb is present in 42% to 65% recreational cyclists.^[2,3]^ Even in the professional cyclists, overuse injuries are more frequent in the lower extremities.^[4]^ Indeed, De Bernardo et al^[4]^ found that 51.5% of total injuries in top-level professional road cyclists are represented by overuse conditions and that, among them, 67.7% afflict lower limbs; 26.4% of these lower limbs injuries are represented by hamstrings and quadriceps contractures. Muscle contracture is an involuntary, painful, and persistent contraction of 1 or more skeletal muscles, which represents a defensive action occurring when the muscle is stressed beyond its physiological tolerance.^[5]^

The evaluation of the contraction state, be it basal or not, of a given muscle can be achieved measuring specific mechanical parameters reflecting its viscoelastic properties, namely muscle tone and stiffness.^[6–8]^

According to De Bernardo et al^[4]^, elite road cyclists showed a high number of muscle “contractures” presumably due to repetitive eccentric and concentric exercise. “Contractures” are therefore considered to be overuse injuries and the incidence of these types of lesions deserves more attention and should be mentioned in sports injury surveillances.^[4]^ The patient feels an involuntary increase in muscle tone and lack of muscle elasticity during movements.^[5,9]^ Moreover, contracted muscles show increased stiffness, an effect which may cause the onset of more severe damages and pain, particularly after intense training.^[10,11]^ Since it is difficult to address or anticipate an injury in professional sportsman, any strategy to enhance injury prevention represents a sensitive issue in sport medicine. To our best knowledge, very few studies focused on the treatment and prevention of these specific conditions that may include physical, supplementary, and pharmacological support, such as body massage, dietary supplement, and myorelaxant agents.

Thiocolchicoside (TCC) is a semisynthetic sulfur derivative of naturally occurring colchicoside from the seeds of *Gloriosa superba* and *Colchicum autumnale*. TCC has established muscle-relaxant, anti-inflammatory, and antalgic properties,^[12]^ and it has been shown to inhibit the binding of [^3^H] GABA (γ-aminobutyric acid) or [^3^H]strychnine to rat cerebrocortical or spinal cord membranes in vitro and in vivo.^[13–15]^ However, TCC molecular targets and mechanisms of action are not yet completely understood.

TCC is clinically used since more than 45 years for the treatment of muscle pain and contractures in patients with orthopedic, rheumatologic, or musculoskeletal disorders.^[12]^ Its effectiveness in these conditions, particularly in low-back pain, has been shown by several clinical studies^[16–19]^ where TCC had been administered orally, parenterally, and topically. TCC transdermal delivery, which has been studied by Artusi et al^[20]^, has the advantage of avoiding systemic side effects and improving patient compliance. In the form of nongreasy topical foams or gels, particularly when associated to skin penetration enhancers,^[21,22]^ TCC may represent a well tolerated and effective formulation for the treatment and prevention of muscle contractures.

The aim of this observational field study was first, to determine the alterations of tone, stiffness, elasticity, and the level of pain (muscle soreness) of rectus femoris (RF) and biceps femoris (BF) in top class cyclists engaged in 3 multistage races, over a period of 1 week *per* each race; second, to determine whether routine topical TCC administration or any other variable in the management of the athletes, may prevent or reduce the entity of such alterations.

## 2 Methods

### 2.1 General and pharmacological management of the athletes

This is a prospective, observational study carried out in a field setting on 23 top-level cyclists from one of the 17 professional UCI World Tour teams which competed in 3 cycling stage races (Tour de Pologne, Eneco, and Vuelta a España). The protocol of the study has been approved by the Local Ethics Review Committee on May 23, 2015.

Cyclists’ weight and height were measured at the first day of each tour (Pologne, Eneco, and Vuelta a España) and body mass index was calculated; fat mass (FM) and free FM (FFM) were also determined by impedentiometry (Akern BIA 101, Akern Srl, Florence, Italy).

During the competition, all athletes received generic total body massage (every day after each race), according to the standard procedures adopted by the medical staff of the team. In addition to these routine procedures, some athletes received topical treatment with TCC according to the indication of the medical staff. In these athletes (TCC+ group), TCC treatments were performed with a foam containing TCC (Miotens foam, Dompé Farmaceutici S.p.a., Milan, Italy. Miotens foam contains 0.25% TCC; eccipients: Polysorbate 80, propylene glycol, ethyl alcohol, propylene glycol, propylene glycol dipelargonate, benzyl alcohol, sodium mono, and diphosphate, Nerolene lavender, water).

For each of the 3 races, TCC was administered 3 times daily and concomitantly with generic massages (morning prerace, postrace, and postdinner) for 7 days. The amount of foam, about 3/5 mL per muscle group, could slightly vary depending on the extension of the treated area (RF or BF).

No additional pharmacological treatments or supplementary procedures were performed to the athletes in the course of the present study; all the procedures were performed as in routine professional practice and athletes were observed for the entire duration of the study.

During 1-week period for every race the athletes included in the study were evaluated using the Myoton technology (Myoton AS, Tallinn, Estonia) (see below), in order to determine the alterations of muscle tone, stiffness, elasticity, and soreness.

After every race stage (before the dinner) participants were asked to lie in a supine position on a massage table to assess RF, and in a prone position to assess BF. All the assessments were made on the subject's dominant limb in relaxed and isometric contraction state. The entire assessment was carried out by an expert in the use of Myoton technology. For the RF measurement (relaxed state), the subject extended his knee keeping the hip in a neutral position, with sandbags placed on either side of the ankle to maintain this position. Measurements were taken at 2/3 of the distance between the anterior superior iliac spine and the superior pole of the patella, to locate a reproducible site over the muscle belly.^[23]^ The isometric condition of RF was evoked by a hip flexion of 45° and the measurements were carried out by asking the subject to maintain this position. Finally, for the BF measurement, the subject assumed a prone position with his knee extended and hip in a neutral position. The point of measurement was identified after the subject flexed his leg to a 45° angle from the horizontal position, producing a slight isometric contraction of the muscle in order to identify the center of the muscle belly. As in the case of RF, to measure the state of BF isometric contraction subjects were asked to maintain the leg flexed at an angle of 45°.^[24]^

### 2.2 Determination of the best 30-minute power outputs

The best 30-minute power outputs were obtained from the SRM Powermeter (SRM, Julich, Germany) records taken during the 3 races.

### 2.3 Measurements of mechanical properties

Muscle mechanical properties were determined using Myoton technology, an in vivo, noninvasive device capable of measuring the state of tension (non-neural tone) and mechanical properties of individual skeletal muscles, with the advantage of portability and relatively low cost. The device provides objective measurements of 3 mechanical properties of muscles: tone, stiffness, and elasticity,^[7]^ parameters which significantly vary according to the degree of the contracture state of the muscle.

The measurements were made positioning the MyotonPro probe, preloaded at 0.18 N, perpendicularly to the skin over the muscle being assessed, with a precompression of the subcutaneous tissues performed independently of the assessor. The device applied brief (15 ms) low force (0.4 N) mechanical impulses, inducing damped natural oscillations of the underlying tissues. These oscillations were recorded by an accelerometer connected to a friction measurement mechanism in the device. The device then simultaneously calculated the resting tone parameters (frequency of oscillation [Hz]), elasticity of weak oscillations (logarithmic decrement [arbitrary units (AU)], and stiffness [N/m]). Considering the high intraclass correlation coefficients found between 2 10-impulse measurements carried out in the Agyapong-Badu et al^[25]^ study, it was decided that a single 10-impulse measurement was adequate.

### 2.4 Parameters measured

The resting tone, defined as the maximum frequency (F = f_max_) computed from the signal spectrum by fast Fourier transform, was recorded (frequency [Hz]). The higher the frequency of the oscillations (natural oscillation frequency), the greater the muscle tension, which increases with contractions and stretching.^[26]^

Stiffness (N/m) is the measurement of the muscle's ability to resist an external force that modifies its shape^[27]^ and is calculated using the formula: 
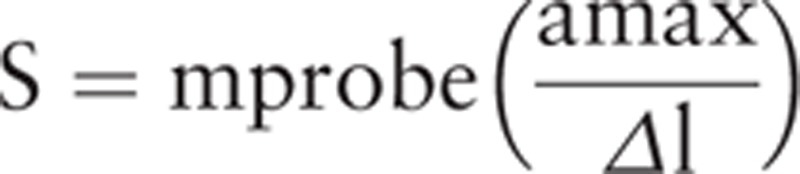


The higher the N/m value is, the greater the muscle stiffness.

Elasticity describes the ability of the tissue to recover its original shape after being deformed. Elasticity can be described with a logarithmic decrement, which shows the dampening of the oscillation, that is, the dissipation of mechanical energy in the tissue in the course of an oscillation cycle. The lower the decrement value is, the smaller the consequent dissipation of mechanical energy, and therefore the greater the elasticity. Elasticity is expressed in AU and represents the logarithm of the dissipation in the tissue during a single oscillation period (log D), calculated as reported elsewhere.^[24,25]^

### 2.5 Muscle soreness

Before the daily determinations of mechanical properties, the level of muscle soreness was assessed using a Likert scale ranging from 0 to 6 (0 = no soreness; 1 = dull feeling of soreness; 2 = light, continuous soreness; 3 = more than light soreness; 4 = annoying soreness; 5 = severe soreness; 6 = intolerable soreness); it was also permitted to report an intermediate value (e.g., 2.5), if necessary.^[28]^ The investigator palpated the medial part of the BF and RF by applying pressure with the tip of 3 fingers (II, III, and IV) for approximately 3 seconds.^[29]^ The participants were instructed to report pain sensation experienced during the palpation. The same investigator assessed the muscle soreness of all participants over race days.

### 2.6 Statistical analysis

Descriptive analyses were performed considering mean standard deviation (SD) variation of tone, elasticity, and stiffness along the race days, for both RF and BF, either in contracted or relaxed conditions.

These descriptive analyses were performed both in the overall group of athletes and in subgroups where differences in the management were identified, namely the athletes that used or not the Miotens foam (TCC+ and No-TCC, respectively). Independent Student *t* test analysis was performed to determine whether these subgroups were homogeneous in terms of age, height, weight FM, and FFM.

Mixed model analysis (MMA) was used for statistical analysis. Dependent variables were tone, elasticity, and stiffness measured using MyotonPro device. All independent variables were considered as fixed effects. In MMA, 4 principal effects and all two-way interactions were considered. MMA was also used for analyzing soreness data in both BF and RF: the day of race and TCC treatment were taken as predictive factors. All elaborations were conducted with α = 0.05. Elaborations and graphics were obtained using Excel (Microsoft) and SPSS version 20.0 (SPSS Inc., Chicago, IL).

## 3 Results

A total of 23 athletes participating to the 3 races were included and evaluated in this real-life study, as described in Table 1. During these races, the recorded maximal mean power for a 30-minute period was 397.6 ± 24 W or 5.4 ± 0.4 W/kg (average ± SD): this value is in keeping with those previously recorded during Tour de France mountain stages^[30]^ and indicates that our subjects can be considered as top level cyclists. All athletes included received the same general massage management. In addition, some of the athletes (TCC+, n = 11) were treated with the topical myorelaxant agent TCC, which is widely used among sport practictioners, while 12 subjects did not receive it (No-TCC). TCC was contained in a nonoil foam (Miotens, with 0.25% of TCC, Dompé Farmaceutici S.p.a.) administered 3 times daily (morning prerace, postrace, and postdinner) for 7 days/race (i.e., the entire duration of Tour de Pologne and Eneco Tour and the first week of race for La Vuelta). The amount of foam was about 3 to 5 mL per muscle group and could slightly vary depending on the extension of the treated area (RF or BF).

**Table 1:**
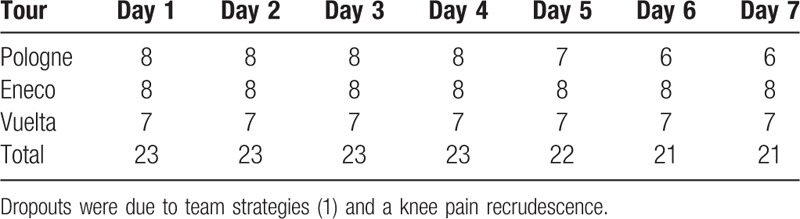
Athletes enrolled in the study.

No additional physical, pharmacological, or dietary supplement treatments were performed to the athletes in the present real-life study.

Therefore, study parameters were analyzed both in the general population included in the study and in the 2 groups of athletes receiving or not TCC (i.e., the only additional variable which has been identified among athletes) in order to assess if TCC may demonstrate any effect on muscle parameters.

Tables 2 and 3 show the baseline characteristics of the included athletes.

**Table 2:**
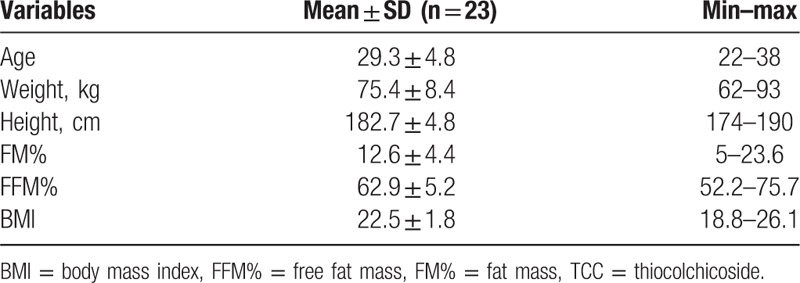
Anthropometric description of the enrolled athletes (mean ± SD).

**Table 3:**
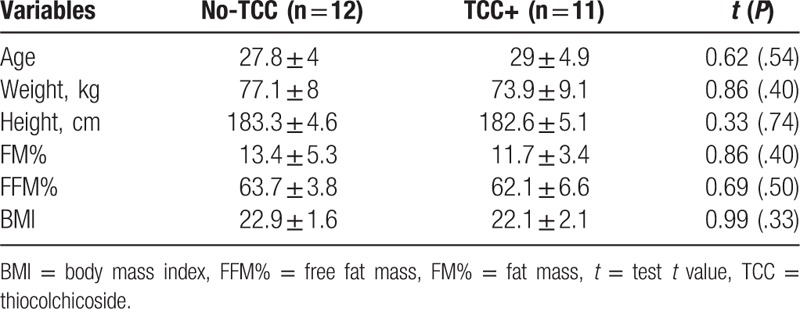
Anthropometric parameters of athletes from No-TCC or TCC+ groups.

As shown in Table 3, there was no significant difference between the major baseline anthropometric parameters of the included athletes.

**Figure 1:**
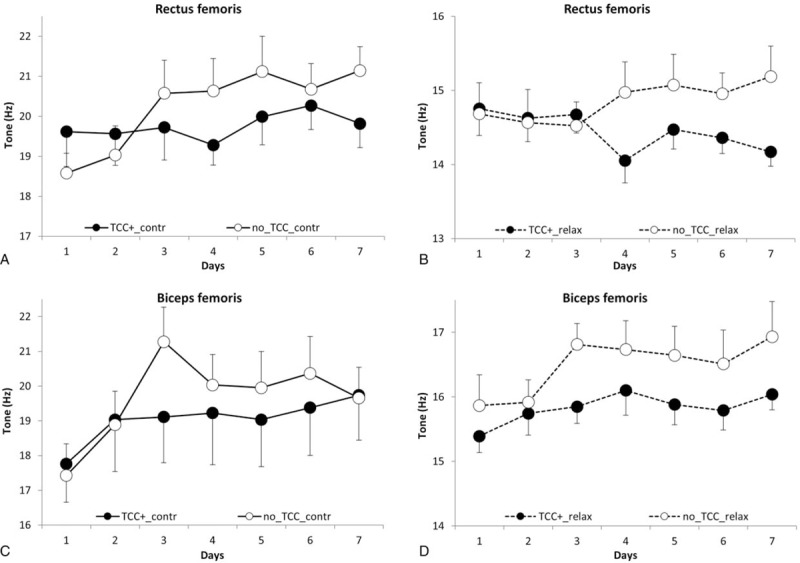
Rectus and biceps femoris tone (Hz) in contracted (A and C) or relaxed (B and D) state as a function of time (race days). Open and closed symbols refer to No-thiocolchicoside (TCC) and TCC+, respectively. Each point represents the mean of the values obtained in days 1 to 7 of the 3 races detailed in the “Methods” section in the 2 groups (No-TCC n = 12; TCC+ n = 11). Bars represent standard errors.

Figures 1, 3, and 4A–D show tone, stiffness, and elasticity values, respectively, in RF and BF as a function of race days in both No-TCC and TCC+ subgroups, either in the contracted (A and C) and relaxed state (B and D).

### 3.1 Tone

Results, analyzed according to MMA, demonstrated that muscle tone increased significantly (*P* = .046) in a time-related fashion, i.e., day-by-day over the races in all the conditions tested and considered (TCC+ and No-TCC, BF and RF, contracted and relaxed state, refer to Fig. 1A–D); the values ranged from an initial value of 16.75 ± 2.83 Hz (the mean of the first determinations in each race), to a final value of 17.82 ± 3.32 Hz (the mean of the last determinations in each race), with a 6.0% increase.

A 27.46% variation (*P* < .001) of tone (BF and RF pooled values) as measured in the contracted (19.62 ± 3.33 Hz) versus relaxed state (15.39 ± 1.47 Hz) was found: such a difference is in accordance with previous reports.^[24]^

With regard to the effects observed in TCC+ group, interestingly, a lower increase of tone (*P* = .012) was found, as compared to No-TCC subjects (Fig. 1A–D). A detailed comparison between the tone values of TCC+ and No-TCC groups is also given in Table 4.

**Table 4:**
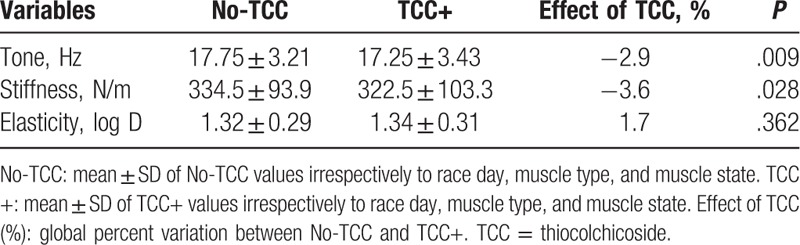
Effects of TCC: comparison between No-TCC and TCC+.

Furthermore, the effect observed in TCC+ as compared to No-TCC group on muscle tone was unrelated to time, that is, race days (*P* = .59), muscle type (*P* = .74), and state (*P* = .97), suggesting that TCC was equally active in RF and BF in both states and in every race day.

**Figure 2:**
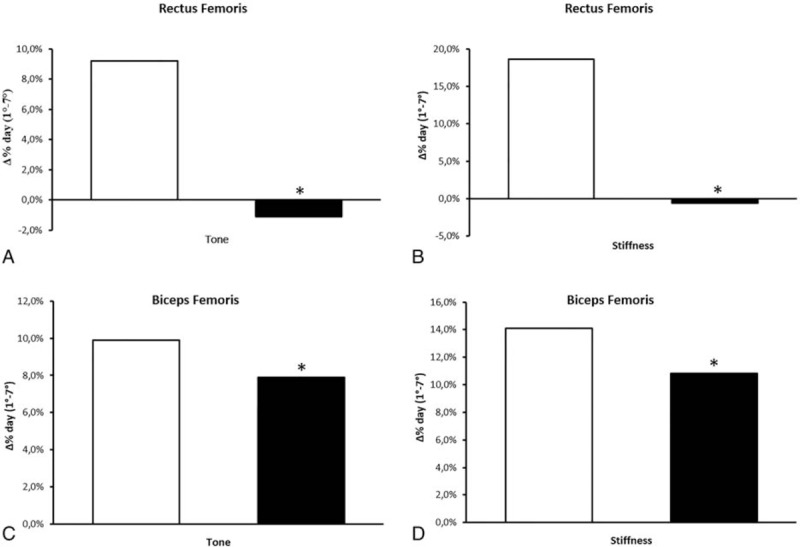
Percent differences between days 1 and 7 (beginning and end of the races) values of tone (A and C) and stiffness (B and D) in No-thiocolchicoside (TCC) (open bars) and TCC+ (solid bars) groups in RF (A and B) and BF (C and D). Percent differences have been calculated using the values obtained pooling the data recorded in the contracted and relaxed states.

The effect of topical TCC treatment can be better appreciated in Fig. 2A and C, showing the percent differences between the tone values (obtained pooling the relaxed and contracted state values) of BF and RF at the beginning and at the end of the races in No-TCC (RF 9.2%; BF 9.9%) versus TCC+ (RF −1.1%; BF 7.9%) groups: smaller differences invariably characterized the TCC+ group.

### 3.2 Stiffness

**Figure 3:**
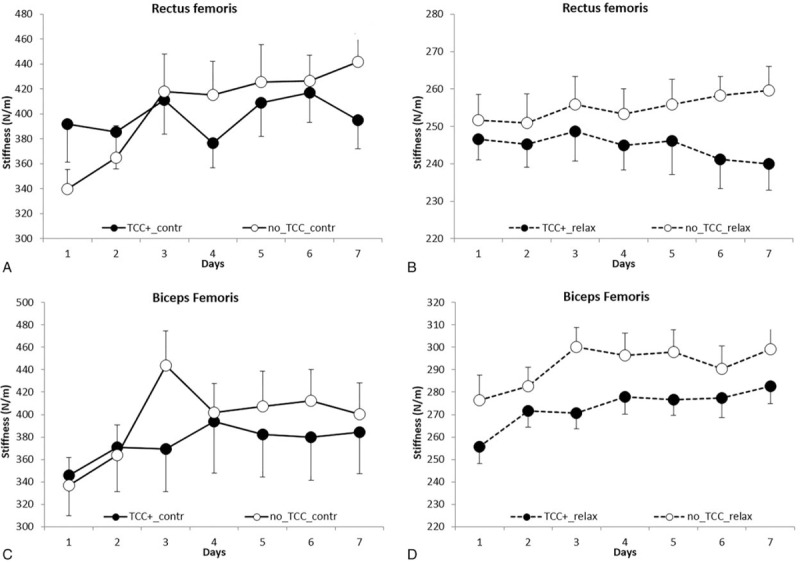
Rectus and biceps femoris stiffness (N/m) in contracted (A and C) and relaxed (B and D) state as a function of time (race days). Open and closed symbols refer to No-thiocolchicoside (TCC) and TCC+, respectively. Each point represents the mean of the values obtained in days 1 to 7 of the 3 races detailed in the “Methods” section in the 2 groups (No-TCC n = 12; TCC+ n = 11). Bars represent standard errors.

Similarly to the tone, muscle stiffness (Fig. 3A–D) increased significantly (*P* = .014) in a time-related fashion, that is, day-by-day over the races in both TCC+ and No-TCC groups; this effect was independent of muscle type and state. Muscle stiffness (BF and RF in both states pooled values) ranged from an initial value of 305.3 ± 84.8 N/m to a final value of 337.1 ± 99.1 N/m, with a 10.5% increase.

Not surprisingly,^[24]^ relaxed state stiffness (BF and RF pooled values) was 265.9 ± 32.4 N/m as compared to 391.2 ± 103.0 N/m in the contracted state, with a +47.1% variation (*P* < .001).

Again, similarly to tone, a significantly lower increase of stiffness (*P* = .028) was observed in TCC+ athletes and in all the conditions tested, as compared to No-TCC subjects (Fig. 3A–D and Table 4).

The effect of TCC can be also appreciated in Fig. 2B and D, which illustrates the percent differences between the stiffness values (BF and RF in both states) at the beginning and the end of the races in No-TCC (RF 18.6%; BF 14.1%) versus TCC+ (RF −0.6%; BF 10.8%): lower differences characterized the latter group.

### 3.3 Elasticity

**Figure 4:**
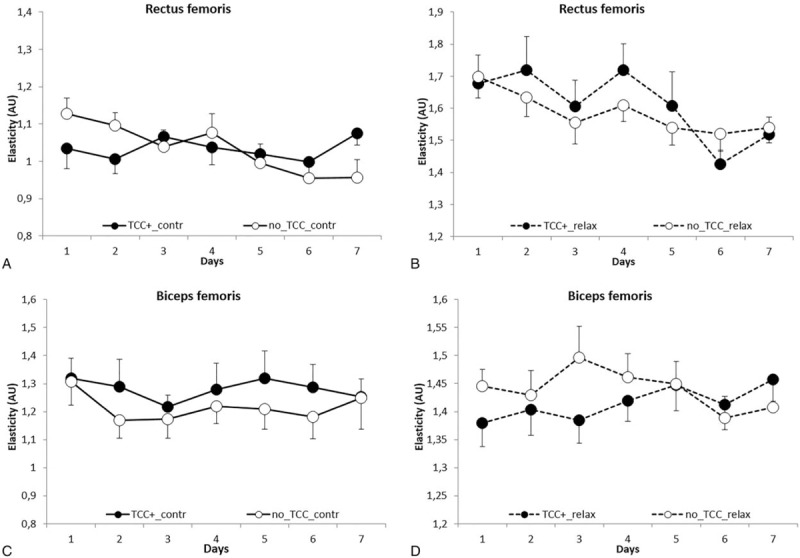
Rectus and biceps femoris elasticity (Ln D) in contracted (A and C) and relaxed (B and D) state as a function of time (race days). Open and closed symbols refer to No-thiocolchicoside (TCC) and TCC+, respectively. Each point represents the mean of the values obtained in days 1 to 7 of the 3 races detailed in the “Methods” section in the 2 groups (No-TCC n = 12; TCC+ n = 11). Bars represent standard errors.

The elasticity changed *quasi*-significantly over time (*P* = .057), in all the conditions tested (Fig. 4A–D); the values (BF and RF in both states pooled values) ranged from 1.37 ± 0.30 at the beginning of the races to 1.31 ± 0.27 AU at the end with a 4.3% variation.

According to data in literature,^[24]^ elasticity significantly (*P* < .001) varied when scored in relaxed or contracted states: AU values were 1.14 ± 0.24 and 1.52 ± 0.22 in the contracted and relaxed state, respectively, with a 24.71% difference.

As to TCC treatment, it did not significantly affect elasticity (*P* = .362); No-TCC subjects showed a mean AU of 1.32 ± 0.29 as compared to 1.34 ± 0.30 in TCC+ group, with a variation of 1.7% (Table 4; Fig. 4A–D).

### 3.4 Muscle soreness

**Figure 5:**
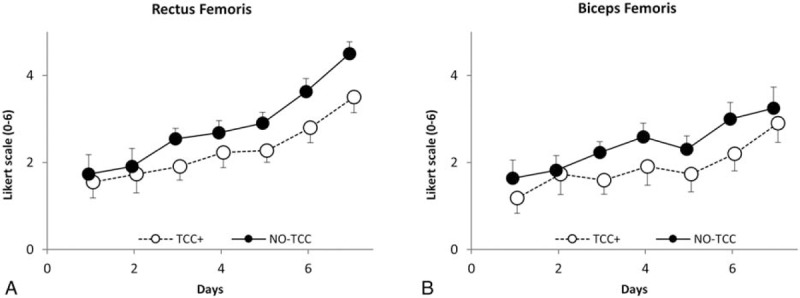
Rectus and biceps femoris soreness as a function of time (race days). Open and closed symbols refer to No-thiocolchicoside (TCC) and TCC+, respectively. Each point represents the mean of the values obtained from days 1 to 7 of the 3 races detailed in the “Methods” section in the 2 groups (No-TCC n = 12; TCC+ n = 11). Bars represent standard errors.

As it could be expected, the results showed a significant increase of muscle soreness (*P* < .001) over time in both groups, in either BF or RF (Fig. 5). Notably, TCC+ athletes exhibited a significantly lower increase in muscle soreness (*P* < .001) in both BF and RF as compared to No-TCC subjects: BF and RF values in No-TCC were 2.35 ± 1.26 and 2.74 ± 1.31, as compared to 1.87 ± 1.36 and 2.26 ± 1.26 in TCC+ group, respectively (Fig. 5A and B).

## 4 Discussion

It is well known that muscle tone and stiffness are the expression of 2 separate components, a neural 1 depending on central nervous system activity and a non-neural, known as myofascial or viscoelastic, component depending on the thixotropic state of the myofascial tissue.

The notion of neural tone, or reflex tone, was derived from experiments on decerebrate animals, which showed markedly exaggerated stretch reflexes. Therefore, the extent of the neural component depends on the intensity of the central nervous system stimuli acting on muscles and is invariably associated with a corresponding, proportional electromyographic activity.

By contrast, the viscoelastic tone of myofascial tissue is not associated with muscle electrical activity, and indeed it cannot be detected with needle or surface electromyography. The viscoelastic tone can be measured exclusively when the muscle is in relaxed state. Moreover, the viscoelastic properties of muscle tissue are not constant, but depend upon previous movements: these movement-dependent changes of the viscoelastic tone are a typical expression of the thixotropic nature of muscles.^[31]^

Data herein presented show that in top-level cyclists tone and stiffness increase as a function of time over the race and that this increase is independent of the state of the muscles (relaxed or contracted) as well as of the type of muscle (RF or BF). Hence, strenuous physical activity causes a prompt increase of both tone and stiffness either in resting, passive conditions, or in isometric voluntary contraction ones.

To our best knowledge, this is the first study carried out in a real-life setting directly dealing with the effects of strenuous, protracted, and repeated physical activity on muscle tone and stiffness in elite cyclists in the course of world tour competitions, demonstrating a time-related, significant increase of these 2 parameters. Although more studies are required in this area, it is conceivable that the increase of tone and stiffness reported herein depends, at least, on 2 different causes, namely (first) chronic muscle overload and (second) psychological distress.

First, chronic muscle overload is known to emerge as a consequence of sustained contraction and repetitive activity usually characterizing the strenuous physical activity required by specific sport disciplines. This muscle condition is thought to be involved in the increased tension and stiffness of myofascial tissue^[32]^; although this increase is poorly documented in the scientific literature, some authors argue that during exercise the calcium ions accumulating in the sarcoplasm of the muscle cell contribute to muscle contractures (see also below). Accordingly, these contractures may be relieved by rest.^[32,33]^

Second, according to some studies, muscle tone is also affected by the so-called unnecessary muscle tension or extraneous muscle tension.^[31]^ Unnecessary muscle tension is thought to depend also on the psychological distress normally associated with increased muscular activity: indeed, according to a recent critical review, there is a direct relationship between excessive worry, mental distress/cognitive load and muscle tension.^[34]^

Both these causes are likely to contribute to the tone and stiffness increase observed in our experimental groups, which are formed by professional elite cyclists participating to a top-level competition.

The third parameter examined, namely muscle elasticity (i.e., the ability of the tissue to recover its original shape after being deformed) did not change significantly over time. However a *quasi*-significant tendency toward increased elasticity was observed, an effect which is the plausible expression of the tone and stiffness variations described above.

Aside from the goal of the present research, it is worth noting that we found a generally higher variability of the above parameters when determined in the contracted as compared to the relaxed state; this observation has also been reported in other studies, such as that by Gavronski et al.^[24]^

Another interesting finding of this study is that we observed for the first time that topical administration of TCC foam during the races significantly reduces the time-related increase of muscle tone and stiffness.

This effect is in line with the locally acting myorelaxant properties of TCC observed by Janbroers^[12]^ in isolated animal muscle preparations or by Ketenci et al^[35]^ in the treatment of acute cervical myofascial pain syndrome. To this regard, since in our study TCC was effective through the transdermal route, it is unlikely that its activity derives from the interactions with central neural pathways, such as glycinergic or GABAergic paths^[13,36]^ as well from a reduction of psychological distress. Rather, TCC effect is more likely dependent on direct actions on the myofascial tissue: these actions are not limited to myorelaxation, but probably include TCC anti-inflammatory and antalgic activities.^[12]^ Accordingly, a lower and significant, time-related increase in muscle soreness was observed in TCC+ group as compared to No-TCC. To this regard it is important considering that inflammation and soreness deriving from particularly stressing muscle utilization, that is, the same situations of the athletes studied herein, are known to result in muscle spasm and contractures.^[35,37]^ Hence TCC is likely to act through multiple mechanisms converging toward the prevention of muscle contractures and their symptoms. In this light, TCC is likely to act in a pleiotropic fashion, a very common mode of action among plant-derived bioactive substances.^[38]^

It is also important to point out that our results have been obtained in a unique situation, that is, a group of 23 top-level, international professional cyclists monitored in the course of a strenuous international championship such as the World Tour Competition. The capacity of TCC, administered topically as foam, to mitigate the increase of tone and stiffness in such an exceptional situation is of particular interest and relevance from the sport medicine point of view. Indeed, reduction of tone and stiffness corresponds to a lesser susceptibility to muscle contractures. In particular, this innovative foam formulation (Miotens), containing propylene glycol and propylene glycol diperlargonate as skin penetration enhancers, has been found to significantly promote TCC accumulation into full human skin thickness in comparison with the simple drug solution^[21]^ and with other commercial TCC-based products.^[22]^

As discussed above, contractures result from overuse-related reversible shortening of the muscle and lead to the so-called delayed-onset muscle soreness. This clinical condition has been related to microscopic muscle fiber damage induced mainly by repeated eccentric muscle contractions. Contractures, also usually referred as to “muscle shortening” or “retractions” among sport practitioners, are a common injury in elite road cyclists, which show a high number of muscle “contractures” caused by repetitive eccentric and concentric exercise.^[4]^ The incidence of these lesions deserves more attention and should be included in sports injury surveillances.^[4]^ Indeed contractures, besides forcing athletes to suspend or limit training and competitions, predispose to more severe muscle and tendon lesions.^[39]^ For example, in elite cycling, riders develop shortening of some muscle groups due to maintained unphysiological positions during cycling: such retractions might have important clinical implications on spine–pelvic muscle imbalance. Hence, any strategy to prevent or reduce the onset of muscle contractures developing in the course of similar stressing situation would deserve greater attention.

A final but not trivial remark is that although we did not specifically address this end-point, TCC does not seem to affect or limit athletes’ performance. This topic will be more thoroughly investigated in future studies. In conclusion, the present study demonstrates that elite cyclists develop increased, time-related tone, stiffness and soreness in BF and RF in the course of top-level competitions, leading to a situation which is a potential threat to their athletic conditions, and that topical TCC foam represents a simple strategy to significantly prevent and attenuate such muscle conditions with the additional advantage of being safe and well tolerated.
